# Dynamic shape remodeling of vesicles by internal active filaments

**DOI:** 10.1016/j.bpj.2025.10.021

**Published:** 2025-10-21

**Authors:** Arash Karaei Shiraz, Amir H. Bahrami

**Affiliations:** 1 Living Matter and Biophysics, UNAM-National Nanotechnology Research Center and Institute of Materials Science & Nanotechnology, Bilkent University, Ankara, Turkey

## Abstract

To interact with their environment, living cells use active cytoskeletal forces to form dynamic membrane structures such as tubular filopodia and sheet-like lamellipodia. To understand the formation and dynamics of these structures, we perform nonequilibrium simulations of dynamically triangulated vesicles under constrained volume. We investigate vesicle shape remodeling driven by local effects of internal active filaments, as well as large-scale shape transformations resulting from volume changes controlled by the osmotic effect. We identify the morphological behavior of vesicles across varying volumes and filament properties, including concentration, mobility, stiffness, and length. Our simulations reveal dynamic, unstable vesicle structures—such as branched tubes, sheet-tubes, cup-tubes, and compartmentalized vesicles—composed of tubular, sheet-like, and cup-like segments. These structures continuously reorganize, interconverting between different shape components while maintaining nearly constant proportions. In particular, unstable branched tubes form under low vesicle volume and low filament mobility. Remarkably, their restructuring accelerates as filament mobility decreases, suggesting that their dynamics are primarily governed by global vesicle remodeling under constrained volume. Notably, branched tubes arise only in the presence of active filaments and vanish when filaments become apolar due to shortening and loss of anisotropy. Our findings reveal novel nonequilibrium pathways for generating unstable, dynamic cellular structures such as branched tubes, sheet-tubes, and compartmentalized vesicles. These insights not only advance our understanding of complex organelle morphologies and cellular protrusions but also suggest new mechanisms for actively shaping synthetic membrane systems.

## Significance

Dynamic shape transformations of cellular membranes, actively driven by the cytoskeleton, are central to key biological processes such as motility, division, and endocytosis. Using a minimal model of a vesicle containing internal active filaments, we computationally explore how cytoskeletal activity remodels membrane shape far from equilibrium. Our simulations reveal a rich spectrum of unstable hybrid structures—such as branched tubes and sheet-tubes—that dynamically interconvert their shape components. These findings offer a physical basis for understanding unstable membrane morphologies, including branched tubular networks and protrusive structures like filopodia and lamellipodia. By uncovering how internal filament activity generates and maintains complex membrane geometries, our work provides new insight into cytoskeleton-driven membrane remodeling and informs the design of shape-changing synthetic cells.

## Introduction

Active matter systems describe collections of energy-consuming agents that convert chemical energy into motion, giving rise to emergent behaviors such as swarming, pattern formation, and self-organization (1,2,3). These systems span synthetic active colloids, vibrated granular rods, and biological entities such as bacterial colonies, cytoskeletal filaments, and cells. A particularly important class is biological active matter, where nonequilibrium forces generated by molecular motors and polymerizing filaments organize the interior of cells and drive large-scale cellular dynamics (4,5).

In living cells, the cytoskeletal network exerts active forces that dynamically remodel membranes. This active membrane remodeling underlies essential processes including cell division (6,7,8), migration through lamellipodia and filopodia (9,10,11,12), cytoplasmic streaming (13), and membrane engulfment during endocytosis and phagocytosis (14,15). Other examples include the formation of tethers and blebs (16,17) and branched morphologies such as neuronal dendrites (18,19). Understanding how active internal elements sculpt membranes remains a central challenge in cell biophysics.

To address this complexity, bottom-up approaches in synthetic biology model cells using lipid vesicles encapsulating active matter components (16,20,21,22,23) or active droplets (24,25). Experiments have shown that such systems can adopt highly dynamic morphologies: encapsulated microtubules and kinesin motors deform vesicles into oscillatory and rotating shapes (21), actomyosin networks drive membrane tension fluctuations (16), and motile colloids or active droplets produce nonaxisymmetric shapes (22,24,26). These biomimetic models reveal how internal active stresses couple to membrane mechanics to generate emergent dynamics.

Complementary simulations have examined active vesicles containing isotropic self-propelled particles or anisotropic filaments. Isotropic active particles can generate pearled tubes (27), induce shape fluctuations (28,29), and promote transitions between oblate, prolate, and stomatocyte morphologies (22,30,31). Anisotropic active filaments, which align with membrane curvature and exert directional forces, can drive tubulation and complex deformations (26,32,33). Although these studies have mapped equilibrium or steady-state shapes, most neglect global constraints such as fixed volume or do not explore dynamic remodeling. In particular, little is known about how active filaments reorganize vesicles dynamically under varying volume/area conditions.

Shape transformations in active vesicles arise from the interplay between global, large-scale deformations and local, short-range remodeling. Local effects, such as the swim pressure of internal active particles, directly induce membrane protrusions and indentations (27,34,35). In contrast, global constraints—such as changes in volume/area ratio, controlled by osmotic conditions or membrane growth—indirectly shape vesicles by altering their geometry (36). Similar effects have been observed in bulk active systems (37). Membrane-mediated alignment of anisotropic active elements can further drive global deformations (32,38), and recent experiments show that internal cytoskeletal networks can generate traveling shape waves in synthetic vesicles (39).

The resulting vesicle morphologies depend on membrane elasticity, spontaneous curvature, particle-membrane interactions, and the volume/area ratio. For active elements, propulsion strength, concentration, and filament flexibility and anisotropy are key factors (30,32,40). Prior work has examined shape fluctuations of active membranes (28,29) and vesicle transformations driven by isotropic particles—with or without adhesion (22,27,28,30,31)—and by anisotropic filaments (26,32,33), including in two-dimensional models (26,41,42,43). However, these studies mostly focus on steady-state shapes. Dynamic remodeling—particularly at low volume/area ratios—remains poorly understood, with the exception of pearled tubes whose compartment number fluctuates due to internal activity (27). A comprehensive understanding of how global constraints and local active forces combine to drive nonequilibrium structures and their dynamic rearrangement is still lacking.

Here, we explore nonequilibrium dynamic shape transformations of elastic vesicles containing self-propelled, semiflexible filaments. Using overdamped Langevin dynamics simulations of dynamically triangulated membranes, we systematically vary filament mobility, length, flexibility, and concentration, along with the reduced volume of the vesicle, which represents the volume/area ratio in our model. We identify a rich variety of dynamically remodeling morphologies, including transient sheets, tubes, and cups (which can also appear as stable equilibrium shapes (44,45,46)), as well as unstable hybrid structures such as sheet-tubes, branched tubes, and pearled tubes. In contrast to previous simulations where internal filaments led to steady-state shapes under unconstrained volumes (32), we observe persistent structural rearrangements driven by filament activity and confinement. These dynamically rearranging structures typically combine basic stable membrane shapes—sheets, cups, and tubes—into unstable hybrid morphologies that continuously interconvert while retaining the relative proportions of each section. Prime examples include branched tubes and sheet-tubes. At low volume/area ratios, vesicles form highly dynamic, branched tubular networks, with branches that emerge and retract at rates proportional to filament mobility and concentration. Interestingly, lower filament concentrations promote greater reorganization. Our results further demonstrate that filament anisotropy—tuned via length and flexibility—plays a central role in determining both morphology and dynamic behavior.

## Materials and methods

Our model is composed of dynamically triangulated vesicles and active filaments that move inside the vesicles under external active forces.

### Active filaments

Active filaments are modeled as semiflexible polymer chains composed of beads that undergo passive Brownian motion. Each two beads belonging to different filaments experience a repulsive force with the interaction potential given by

(1) $$U_{bb}\left(r\right)=\frac{1}{2}k_{bb}{\left(r-\sigma \right)}^{2}H\left(\sigma -r\right)\text{,}$$

where *r* is he distance between the beads, *k*_*bb*_ is the potential coefficient adjusting the repulsion strength, *σ* denotes the effective bead diameter, and *H* is the Heaviside step function (27). Each filament consists of *N*_*bf*_ beads, which are connected by *N*_*bf*_-1 links through applying a harmonic bond potential,

(2) $$U_{fbond}\left(r\right)=\frac{1}{2}k_{fb}{\left(r-r_{0}\right)}^{2}\text{,}$$

between every two consecutive beads inside the filament. The bond stiffness, *k*_*fb*_, and the equilibrium bond length *r*_0_ control the bond lengths of the filaments. The filament length is defined as $L=\left(r_{0}\left(N_{bf}-1\right)+\sigma \right)/\sigma$. Bending deformations of the filaments are governed by a bending potential, given as

(3) $$U_{fbend}\left(r\right)=\frac{1}{2}\kappa_{f}{\left(\theta -\pi \right)}^{2}\text{,}$$

with *k*_*f*_ representing the filament bending stiffness and *θ* denoting all the *N*_*bf*_-2 angles formed between each three consecutive beads in each filament. The persistence length P characterizes the effective end-to-end filament distance, rescaled by *σ*. A total number of *N*_*f*_ filaments in vesicles results in a filament concentration, *ϕ* = *N*_*f*_*N*_*bf*_(*σ*/2*R*)^3^, representing the volume fraction of a spherical vesicle with surface area *A* that is occupied by all filaments. Interactions between the filaments and the vesicle are governed through repulsive forces acting between all filament beads and the vesicle nodes (vertices) given by

(4) $$U_{bv}\left(r\right)=\frac{1}{2}k_{bv}{\left(r-\sigma_{bv}\right)}^{2}H\left(\sigma_{bv}-r\right)\text{,}$$

where *r* is the bead-vertex distance, *k*_*bv*_ = *k*_*bb*_ is the potential coefficient adjusting the repulsion strength, and *σ*_*bv*_ = *σ* denotes the effective bead-vesicle distance.

Filament displacement is governed by the motion of filament beads with a position vector **r**_*i*_(*i* = 1…*N*_*bf*_) following the Langevin equation,

(5) $$m_{b}\;{\ddot{r}}_{i}=\nabla_{i}U_{btot}-\gamma \;{\dot{r}}_{i}-\sqrt{2k_{B}T\gamma }\xi_{i}\left(t\right)+f_{p}\frac{r_{i+1}-r_{i}}{\left|r_{i+1}-r_{i}\right|}\text{,}$$

where *U*_*btot*_ is the sum of all interaction potentials acting on beads, *m*_*b*_ is the bead mass, $\dot{r_{i}}$ and $\ddot{r_{i}}$ are the first and the second time derivatives of the position vector **r**_*i*_ of bead *i*, and ∇ is the spatial derivative at the position vector of bead *i*. Here, *γ* represents the effect of the surrounding viscous fluid on the filaments, and ***ξ***_*i*_(*t*) is a Gaussian white noise with its Cartesian components following ⟨*ξ*_*i*_(*t*)⟩ = 0 and ⟨*ξ*_*i*_(*t*)*ξ*_*j*_(*t*′)⟩ = *δ*_*ij*_*δ*(*t*-*t*′) representing thermal fluctuations. The last term in Eq. (5) represents the external active force of magnitude *f*_*p*_, applied to filament beads along the filament. The active force in our model is introduced phenomenologically, following standard practice in active matter simulations. Although biological systems balance such forces via cytoskeletal structures, in our case, the membrane provides the necessary counterforce, maintaining momentum conservation.

Langevin simulations were performed for 50 s and, in some cases, extended to 100 s. Data was collected every 0.01 s, and the last 30 s of each simulation was used to calculate the mean and standard deviation of various properties.

### Triangulated membrane model of the vesicles

The vesicles in our model are presented by a three-dimensional triangulated membrane composed of *N*_*v*_ point vertices or nodes connected by *N*_*l*_ links, which together form *N*_*t*_ = 2(*N*_*v*_-2) triangles (47). Membrane bending is governed by the Helfrich model of curvature elasticity (48), according to which the bending energy of the vesicle, with negligible spontaneous curvature, *C*_0_ = 0, is given by

(6) $$U_{bend}=2\kappa \;\oint_{A}M^{2}dA\text{,}$$

where *M*=(*C*_1_+*C*_2_)/2 is the mean of the two principal curvatures, *C*_1_ and *C*_2_, (and *A* is the vesicle area. All our simulations are performed in constant *v* ensemble, where the fixed vesicle area, *A* = *A*_0_, identical for all vesicles, is preserved using a harmonic potential given by

(7) $$U_{A}=\frac{k_{A}{\left(A-A_{0}\right)}^{2}}{2A_{0}}\text{,}$$

where *k*_*A*_ is the area conservation coefficient. To generate vesicles with given values of *v* for each simulation, the vesicle volume *V* is also constrained to remain nearly constant at target values *V*_0_ using the harmonic potential,

(8) $$U_{V}=\frac{k_{V}{\left(V-V_{0}\right)}^{2}}{2V_{0}}\text{,}$$

where *k*_*V*_ is the volume conservation coefficient. The links of the triangulated vesicles experience a bond potential that controls the link length. This potential consists of a repulsive core (*U*_*r*_) and an attractive tail (*U*_*a*_), each taken to have a half-harmonic form given by the following:

(9) $$U_{r}\left(r\right)=\left\{ \begin{array}{cc} \frac{1}{2}k_{b}{\left(r-r_{r}\right)}^{2} & \text{if}\;r<r_{r}\\ 0 & \text{if}\;r\geq r_{r} \end{array}\right.$$

(10) $$U_{a}\left(r\right)=\left\{ \begin{array}{cc} 0 & \text{if}\;r\leq r_{a}\\ \frac{1}{2}k_{b}{\left(r-r_{a}\right)}^{2} & \text{if}\;r>r_{a}, \end{array}\right.$$

where *k*_*b*_ is the bond stiffness, and *r*_*a*_ and *r*_*r*_ are the potential cutoff lengths expressed in terms of *l*_0_ (see Table S1). Here, *l*_0_ denotes the side length of identical equilateral triangles whose total area equals the reference vesicle area *A*_0_. In our simulations with relatively low Pe ≤ 15, these half-harmonic potentials keep the lengths of the vesicle edges withing a range comparable to similar bond potentials.

### Membrane-membrane interactions

Small vesicle volumes typically lead to the formation of long, narrow tubes, thin sheets, and thin cup-like structures, where distant membrane segments may come into close proximity. In particular, opposing bilayers are found in close contact in sheet-like and cup-like morphologies. Additionally, different sections of a single long tube or two adjacent tubes in a branched structure may come into close proximity. Although the latter case (tubular membranes) does not pose computational issues, intersecting apposing membranes lead to an underestimation of *v*. To prevent this, we apply a pairwise interaction potential, *U*_*vv*_(*r*) = *U*_*bv*_(*r*) (Eq. (4)), between each pair of nonneighboring vertices of the vesicle. This computationally expensive interaction potential prevents membrane self-intersection, ensuring an accurate calculation of *v* and maintaining consistency with actual membranes, which do not exhibit self-intersection.

The sum of all these interaction potentials, along with *U*_*bv*_, which represents bead-vertex repulsive forces, and *U*_*vv*_, which prevents membrane self-intersection, gives rise to the total interaction potential, *U*_*vtot*_. The vesicle dynamics is modeled by the Langevin equation:

(11) $$m_{v}{\ddot{r}}_{i}=\nabla_{i}U_{vtot}-\gamma {\dot{r}}_{i}-\sqrt{2k_{B}T\gamma }\xi_{i}\left(t\right)\text{,}$$

where *i* = 1…*N*_*v*_ runs over all vertices, *m*_*v*_ is the vertex mass, and other parameters are defined similar to Eq. (5) for filament beads. A Verlet algorithm was used to integrate equations of motion, Eqs. (5) and (11).

### Membrane fluidity

The triangulated vesicle model introduced so far represents a vesicle as a three-dimensional network with a fixed connectivity between the network nodes as vesicle vertices. The network connectivity does not allow the vertices to freely move inside the vesicle plane. Actual membranes are however fluid in the sense that lipid molecules can easily diffuse inside the membrane plane. To fulfill membrane fluidity the network connectivity needs to be broken regularly in order to allow vertices to freely diffuse in the vesicle membrane. This is done by flipping the vesicle bonds with a time frequency *ω* that replaces the two triangles sharing the edge by two new triangles. During flipping, all vesicle bonds are flipped by a probability *q* where the flipping acceptance is decided by a Monte Carlo algorithm. The total change in the vesicle energy is calculated as the sum Δ*U* = Δ*U*_*bend*_ + Δ*U*_*A*_ + Δ*U*_*V*_ + Δ*U*_*r*_ + Δ*U*_*a*_ upon bond flipping. Bond flipping is then always accepted if it is energetically favorable with Δ*U* < 0 and otherwise with the probability of *exp*[−Δ*U*/(*k*_*B*_*T*)].

### Vesicle shape

To distinguish vesicle shapes, we use a vesicle shape index, *α* = Δ*a*/Δ*a*_*T*_, which is calculated from the membrane asymmetry,

(12) $$\Delta a=\frac{1}{2\sqrt{\pi A}}\oint MdA\text{,}$$

of the vesicle and the membrane asymmetry Δ*a*_*T*_ of a cylindrical tube at the same volume. To estimate Δ*a* for sheet-like and tubular vesicles at each *v*, we use simplified axisymmetric cylindrical tube and toroidal sheet structures, as shown in Fig. S7 *A*. The cylindrical tube is modeled as a straight cylinder of radius *r*_*T*_ and length *L*_*T*_ = *br*_*T*_, capped by two spherical caps at both ends (Fig. S7 *A*, right). Its surface area and volume are given by

(13) $$A_{T}=2\pi r_{T}^{2}\left(b+2\right)\text{,}$$

and

(14) $$V_{T}=\pi r_{T}^{3}\left(b+\frac{4}{3}\right)\text{,}$$

leading to a reduced volume,

(15) $$v_{T}=\frac{6\sqrt{\pi }V_{T}}{A_{T}^{3/2}}=\frac{3\left(b+\frac{4}{3}\right)}{\sqrt{2}{\left(b+2\right)}^{3/2}}\text{.}$$

**Figure 1:**
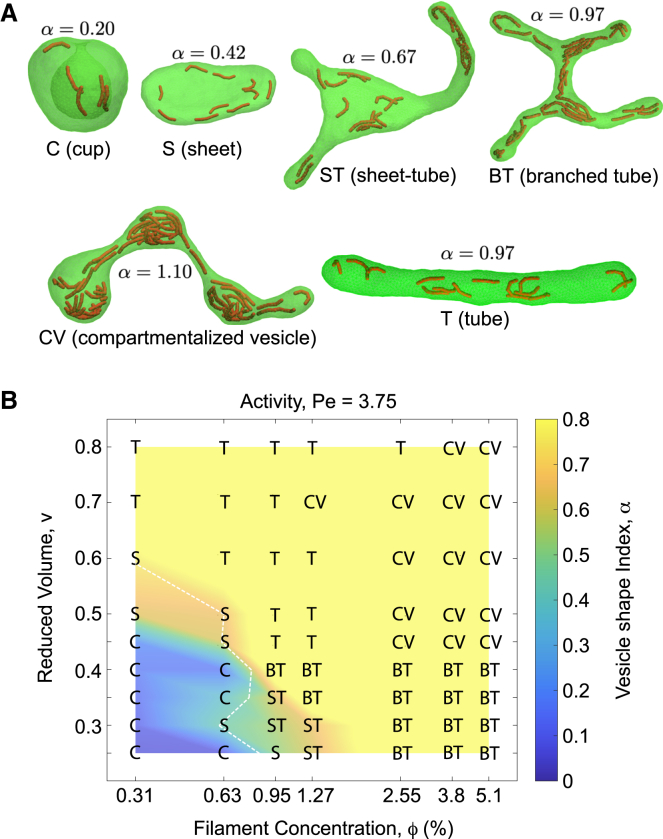
Vesicle morphologies formed by internal active filaments. (*A*) Active vesicles either adopt one of the fundamental vesicle shapes—tubes (Ts), sheets (Ss), or cups (Cs)—which are also observed at equilibrium—or form hybrid structures such as sheet-tubes (STs), branched tubes (BTs), and compartmentalized vesicles (CVs) including pear-shaped vesicles and pearled tubes. (*B*) Morphology diagram of active vesicles with varying *ϕ* and *v* at a fixed activity Pe = 3.75 for relatively stiff filaments (*χ* = 25) of length (L=6.12). Vesicle shapes are characterized by the shape index *α*, which is based on membrane asymmetry (Δ*a*) and is color-mapped onto the shape diagram. The white dashed line indicates toroidal sheets predicted by the theoretical vesicle model at different values of *v* (see materials and methods).

The membrane asymmetry of the tube is obtained from

(16) $$\Delta a_{T}=\frac{1}{2\sqrt{\pi A_{T}}}\oint MdA=\frac{b+4}{\sqrt{8\left(b+2\right)}}\text{,}$$

where we use *M* = 1/*r*_*T*_ (*C*_1_ = *C*_2_ = 1/*r*_*T*_) for the spherical caps and *M* = 1/(2*r*_*T*_) (*C*_1_ = 0, *C*_2_ = 1/*r*_*T*_) for the cylindrical section. We then use Eqs. (15) and (16) to express Δ*a*_*T*_ as a function of *v*, which in turn allows us to determine *α* for any vesicle shape at a given *v*. Table 1 presents the different values of *v* used in our simulations, along with the corresponding values of Δ*a*_*S*_ and Δ*a*_*T*_, given by the left and right curves in Fig. S7 *B* for sheet-like and tubular vesicles, respectively. The left curve in Fig. S7 *B*, which corresponds to toroidal sheets, is shown as white dashed lines in Figs. 1 *B*, 4 *A*, 5, S2, and S5.

**Table 1:** Membrane asymmetry of sheets and tubes versus reduced volume

*v*	0.25	0.3	0.35	0.4	0.45	0.5	0.6	0.7	0.8
Δ*a*_*S*_	1.083	1.078	1.072	1.067	1.061	1.056	1.044	1.033	1.022
Δ*a*_*T*_	3.06	2.57	2.22	1.96	1.77	1.61	1.39	1.23	1.13

To demonstrate that *α* obtained in this way serves as an accurate measure for defining vesicle shape, we introduce a simplified axisymmetric sheet vesicle model. This shape consists of a toroidal surface with radius of curvature *r*_2_, enclosed by two circular planes of radius *r*_1_ = *cr*_2_ on both sides (Fig. S7 *A*, left). The area and volume of this sheet are given by

(17) $$\begin{array}{cc} A_{S} & =2\pi c^{2}r_{2}^{2}+2\pi \int_{0}^{\pi }Xr_{2}\;d\psi \\ & =\pi r_{2}^{2}\left(2c^{2}+2\pi c+4\right) \end{array}$$

and

(18) $$\begin{array}{cc} V_{S} & =\int_{0}^{\pi }\pi X^{2}\;\sin\;\psi \;r_{2}\;d\psi \\ & =\pi r_{2}^{3}\left(2c^{2}+\pi c+\frac{4}{3}\right)\text{,} \end{array}$$

where *ψ* is the angle between the tangent line to the planar sheet contour and the horizontal axis, and *X* = *r*_1_+*r*_2_*sinψ* is the radial distance of a point on the contour form the axis of symmetry (44,45,49) (Fig. S7 *A*, left). This leads to a reduced volume,

(19) $$v_{S}=\frac{6\sqrt{\pi }V_{S}}{A_{S}^{3/2}}=\frac{6\left(2c^{2}+\pi c+\frac{4}{3}\right)}{{\left(2c^{2}+2\pi c+4\right)}^{3/2}}\text{.}$$

The membrane asymmetry of the sheet is obtained as follows:

(20) $$\Delta a_{S}=\frac{1}{2\sqrt{\pi A_{S}}}\oint MdA$$

$$=\sqrt{\frac{\pi }{A_{S}}}\int_{0}^{\pi }\left[\frac{1}{2r_{2}}+\frac{\sin\;\psi }{2X}\right]Xr_{2}d\psi$$

$$=\frac{\pi c+4}{2{\left(2c^{2}+2\pi c+4\right)}^{1/2}}\text{,}$$

where *dA* = 2*πXds* = 2*πXrdψ* is expressed in terms of the arc length *s* on planar membrane contour, as shown in Fig. S7 *A*, left. Eqs. (19) and (20) are used to compute Δ*a*_*S*_ at each *v*, which is then used to determine *α* for the sheet vesicle, as indicated by the left curve in Fig. S7 *B* and the white dashed lines in Figs. 1 *B*, 4 *A*, 5, S2, and S5.

### Membrane tension

We calculate membrane tension using the virial expansion (40,50). The virial contribution from the global area and volume constraints is given by

(21) $$V_{av}=\sum_{\alpha }\left\langle f_{i}^{av,\alpha }r_{i}^{\alpha }+f_{j}^{av,\alpha }r_{j}^{\alpha }+f_{k}^{av,\alpha }r_{k}^{\alpha }\right\rangle \text{,}$$

where $f_{i,j,k}^{av}$ are the constraint forces acting on the vertices *i*, *j*, and *k* of a triangular membrane element, and *α* = *x*,*y*,*z* denotes the Cartesian coordinates. The virial contribution from the elastic bond forces *f*^*b*^ acting on the vesicle edges (links) is

(22) $$V_{b}=\sum_{\alpha }\left\langle f_{i}^{b,\alpha }r_{i}^{\alpha }+f_{j}^{b,\alpha }r_{j}^{\alpha }\right\rangle \text{.}$$

The total virial contribution from all forces on each vertex is then given by *V* = *V*_*av*_/3 + *V*_*b*_/2. Membrane tension is calculated via spatial and temporal averaging of the local stresses:

(23) $$\bar{\lambda }={\left\langle \frac{1}{2A_{i}}\left(V_{i}\left(t\right)+2k_{B}T\right)\right\rangle }_{i,t}\text{,}$$

where *V*_*i*_(*t*) is the total virial contribution at vertex *i* at time *t*, and *A*_*i*_ is the area assigned to vertex *i*, approximately one-third of the total area of the surrounding triangles. The factor of two accounts for the two-dimensional nature of the membrane. The kinetic contribution 2*k*_*B*_*T* is estimated from the equipartition theorem. Contributions from the bending energy are neglected, as the associated forces act perpendicular to the membrane surface and do not contribute to in-plane tension.

## Results and discussion

### Physical parameters

The morphological behavior of nonequilibrium vesicles with constrained volume and internal active filaments is governed by a combination of filament and vesicle parameters, including the vesicle volume/area ratio, filament mobility (activity), concentration, and anisotropy, which is determined by filament length and bending stiffness. These factors are captured by the following dimensionless parameters: the reduced volume $v=6\sqrt{\pi }V/A^{3/2}$, where *A* and *V* are the vesicle surface area and volume, respectively; the Péclet number $\text{Pe}=\sigma f_{p}/\left(k_{B}T\right)\in \left[3.75,15\right]$, which controls filament mobility; the filament concentration *ϕ*; the rescaled filament length or aspect ratio L; and the dimensionless filament bending stiffness *χ* = *κ*_*f*_/*κ*, where *κ* and *κ*_*f*_ are the membrane and filament bending rigidities, respectively.

In our model, active filaments are represented as semiflexible polymer chains composed of spherical beads of diameter *σ*, connected via harmonic bonds. Each bead experiences a propulsion force *f*_*p*_ applied tangentially along the chain axis, i.e., along the bond directions between adjacent beads (32,51). This approach is widely used and remains valid even for highly flexible filaments, despite noisier bond directions at low rigidity (52). For a given number of filaments *N*_*f*_, the concentration *ϕ* is defined as the volume fraction of a spherical vesicle at *v* = 1 that is occupied by all filament beads. The propulsion force determines the Péclet number, $\text{Pe}=\sigma f_{p}/\left(k_{B}T\right)$, which quantifies the relative strength of active propulsion to thermal fluctuations and thereby controls filament mobility and activity level. In our simulations, filaments consist of only a few beads, with spacing comparable to that between membrane vertices, making their length much smaller than the vesicle diameter.

The vesicle is modeled as a fluid, triangulated membrane consisting of mobile vertices and dynamic edges, which are flipped to maintain membrane fluidity. All simulations are performed in a constant-area ensemble, with total membrane area *A*_0_ = 4*πR*^2^ setting the characteristic size scale *R*. The vesicle volume is constrained to achieve a prescribed reduced volume *v*, representing the effective volume/area ratio. See materials and methods for full details of the simulation setup and parameters.

### Nonequilibrium morphologies of active vesicles

We find that nonequilibrium vesicles exhibit complex dynamic morphologies, adopting either one of the stable vesicle shapes—tubes, sheets, or cups, which also appear in equilibrium systems (44,45)—or hybrid forms that combine these structures (Fig. 1 *A*). The unstable hybrid shapes include sheet-tubes, which consist of sheet-like regions with protruding tubular segments; branched tubes, featuring three-way tubular junctions; and compartmentalized vesicles, comprising either pear-shaped vesicles or partially constricted morphologies forming dynamically interconnected compartments (Figs. 1 *A* and S1).

To quantify these shape transformations, we use Δ*a*, defined as the surface integral of the membrane mean curvature, which serves as a measure of membrane asymmetry in theoretical and triangulated membrane models (44,46); see materials and methods for details. Although Δ*a* was originally introduced in the context of lipid bilayers with distinct leaflets, it has also been applied to single-layer models as a geometric quantity independent of molecular details. For a given reduced volume *v*, Δ*a* assumes characteristic values for pure shapes: Δ*a*_*C*_ for cups, Δ*a*_*S*_ for sheets, and Δ*a*_*T*_ for tubes, with the general ordering Δ*a*_*C*_ < Δ*a*_*S*_ < Δ*a*_*T*_ across all *v* (44,46). Hybrid shapes yield intermediate values of Δ*a* that reflect the proportions of their constituent parts. For instance, sheet-tubes exhibit Δ*a* values between Δ*a*_*S*_ and Δ*a*_*T*_, depending on the relative extent of the tubular and sheet-like regions. To normalize this geometric measure, we define a dimensionless shape index, *α* = Δ*a*/Δ*a*_*T*_, which takes small values (*α* ≪ 1) for cups, intermediate values for sheets, reaches *α* ≈ 1 for tubular vesicles and branched tubes, and exceeds unity (*α* > 1) for pearled tubes. This index serves as a useful proxy to distinguish hybrid morphologies, particularly in dynamic regimes. Specific values of Δ*a* for different shapes and reduced volumes are provided in Table 1; see materials and methods for further discussion.

For a given Pe and fixed filament properties, *χ* and L, the morphological behavior of active vesicles depends on *v* and *ϕ*, as shown in Fig. 1 *B*. The morphology diagram can be divided into four distinct regimes based on vesicle structures. At low values of *v* and *ϕ*, vesicles adopt cup shapes, represented by the lower-left (blue) region of the diagram. Slight increases in *ϕ* and *v* give rise to two distinct regimes within the green region. At low *ϕ*, increasing *v* promotes the formation of sheets (S) in the upper-left part of the green region (brown), which rapidly transition into tubes beyond the white dashed lines representing theoretically predicted toroidal sheets. Further increasing *v* establishes the tubular regime in the top-left part of the diagram, characterized by simple tubes with nearly uniform cross sections.

In contrast, increasing *ϕ* while keeping *v* low drives a rapid transition from cups to sheet-tubes, located in the lower-right brown region of the green area. Interestingly, further increasing *ϕ* transforms these to sheet-tubes into branched tubes with a dynamically varying number of tubular junctions, a regime uniquely observed at low *v* and high *ϕ* in the bottom-right corner of the diagram. Finally, a simultaneous increase in *v* and *ϕ* leads to the fourth regime, located at the top-right of the diagram, predominantly consisting of dynamically reorganizing compartmentalized vesicles with constricted membrane necks separating distinct compartments. White dashed lines in Fig. 1 *B* represent theoretical toroidal sheets at each volume. These lines closely follow the simulated sheet-like vesicles, indicating that *α* serves as a useful measure for distinguishing vesicle shapes. Interestingly, branched tubular networks are observed only at sufficiently small values of *v* ≤ 0.4 and relatively large *ϕ* ≥ 0.95%, whereas larger values of *v* typically give rise to tubes and compartmentalized vesicles. Consistent with previous studies of active vesicle remodeling in the absence of volume constraints (32), we find that filament mobility does not significantly influence the morphological behavior of active vesicle. Although the four vesicle regimes remain similar with slightly shifted boundaries, increasing Pe from 3.75 to 15 decreases the abundance of branched tubes, converting them into tubular vesicles (Fig. S2).

### Dynamic structural reorganization of active vesicles

**Figure 2:**
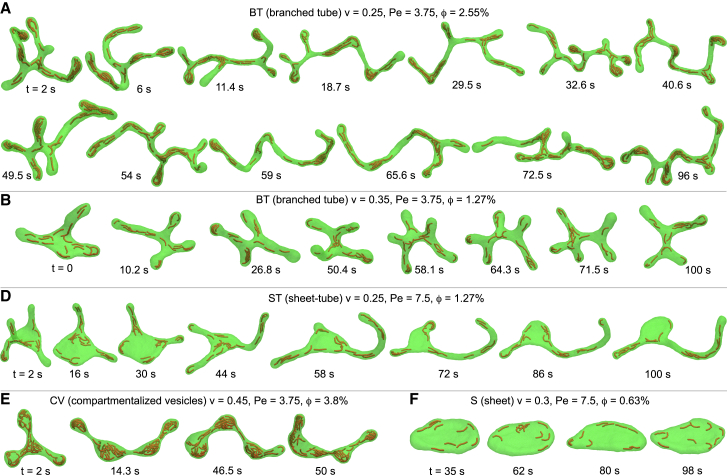
Temporal evolution of dynamically reorganizing active vesicles. Branched tubes with relatively small volumes, (*A*) *v* = 0.25 and (*B*) *v* = 0.35, continuously reorganize their structures by extending and retracting tubes, forming a variable number of three-way tubular junctions. Videos S1 and S2 show real-time animations of (*A*) and (*B*), respectively. (*D*) A dynamic hybrid sheet-tube composed of interconnected sheet-like and tubular sections that interconvert while maintaining a nearly constant fraction of each section over 100 s of simulation. The length of the tubular sections and the area of the sheet-like section remain almost unchanged. Video S3 shows a real-time animation of the sheet-tube structure in (*D*). (*E*) Dynamic evolution of a compartmentalized vesicle, composed of restructuring compartments, at a relatively large *ϕ* over 50 s. (*F*) A sheet with relatively low *ϕ* and *v*, which retains its sheet-like morphology throughout the 100-s simulation, despite continuous structural reorganization.

The active vesicles we observe here dynamically reorganize their structures. In the absence of volume constraint, active vesicles with confined filaments have been shown to exhibit steady-state morphologies (32). In contrast, under constrained volume, we observe that they undergo dynamic shape transformations, particularly evident in unstable hybrid structures such as sheet-tubes, branched tubes, and compartmentalized vesicles. For instance, individual tubes within branched tubular networks emerge and retract dynamically, resulting in a variable number of three-way tubular junctions. This complex dynamic behavior depends on system parameters and is consistently observed across different values of *v* in the branched tubular regime (Figs. 2 *A*, *B* and S3 *A–E*, and Videos S1 and S2. Similarly, the sheet-like and tubular sections of sheet-tubes dynamically interconvert, while maintaining a relatively constant proportion of each (Figs. 2 *D* and S3 *I*, and Video S3). Consequently, the total length of the tubular segments, as well as the tube diameter, remains nearly constant despite fluctuations in the number and individual lengths of the tubes. Comparable dynamic reorganization is observed in compartmentalized vesicles, where both the shape and number of internal compartments fluctuate over time, as well as in pear vesicles with relatively constant proportions of spherical and tubular sections (Figs. 2 *E* and S3 *G*). Even fundamental shapes such as tubes, sheets, and cups exhibit continuous remodeling while preserving their overall geometry, as demonstrated by the temporal evolution of active sheets (Fig. 2 *F*).

**Figure 3:**
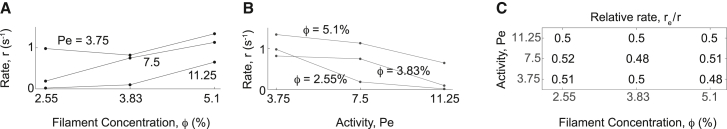
Dynamic reorganization of branched tubes. (*A*) The rate of change in tubular junctions increases with *ϕ* for a fixed Pe, whereas (*B*) it decreases with Pe at a constant *ϕ*. (*C*) All branched tubes exhibit nearly identical emergence and retraction rates, *r*_*e*_ ≈ 0.5, implying that tube emergence and retraction occur with almost equal frequency.

Video S1. Dynamic shape remodeling of a branched tube at low volume Dynamic shape remodeling of the branched tube at v = 0.25 over 100 s, driven by internal active filaments. The video corresponds to the snapshots shown in Fig. 2*A*. At the smallest filament mobility (Pe = 3.75), this branched tube rapidly reorganizes its structure by dynamically varying the number of tubular junctions, as illustrated in the top-left panel of Fig. 3*A*.

Video S2. Dynamic shape remodeling of a branched tube at intermediate volume Dynamic shape remodeling of the branched tube at v = 0.35 over 100 s, as shown in the snapshots in Fig. 2*B*. Compared with the branched tube in Video S1, which has a smaller v, this tube—with the same Pe—exhibits wider diameters and slower dynamic reorganization.

Video S3. Dynamic shape remodeling of a sheet-tube Dynamic shape transitions of a sheet-tube at v = 0.25 over 100 s, corresponding to the snapshots in Fig. 2*D*. Despite continuous interconversion between sheet-like and tubular sections, the area proportion of each component remains nearly constant. Although the number of tubes and their individual lengths fluctuate, both the total tubular length and the tube diameter remain approximately unchanged.

To better understand the dynamic reorganization of active vesicles and its relation to *v* and *ϕ*, we focus on the branched tubular networks, which are particularly prominent at low *v* (Fig. 2 *A* and *B*). The fundamental building block of these networks is a three-way tubular junction, where two tubes intersect, referred to hereafter as a tubular junction. The top-right panel of Fig. 2 *A* shows two such junctions connected to each other to form an H-shaped configuration. Each junction consists of two intersecting tubes with nearly identical diameters. In living cells, similar unstable tubular junctions can emerge through the equilibrium fusion of two membrane tubes (53). In our simulations, they form via the nonequilibrium sprouting of tubular protrusions driven by active forces from self-propelled filaments. Conversely, a tubular junction vanishes when one of the two intersecting tubes retracts. The number of tubular junctions determines the network complexity, whereas its variation over time provides a measure for the network dynamics.

We monitored the temporal evolution of the number of tubular junctions for two representative cases at reduced volumes *v* = 0.25 and *v* = 0.3, as shown in Fig. S4 and Videos S1 and S2, respectively. Rather than averaging over multiple simulation replicas, we performed long-time simulations of 100 s to ensure that the system was sufficiently sampled and the data were statistically meaningful. These trajectories, which contain several events of junction emergence and retraction, are representative and long enough to reliably capture steady-state junction dynamics and stochastic fluctuations. For each *v*, we examined nine combinations of parameters corresponding to three values of (Pe) and (*ϕ*). We then computed the total junction turnover rate *r* = *r*_*e*_ + *r*_*r*_, defined as the sum of the emergence rate *r*_*e*_ and retraction rate *r*_*r*_ of tubular junctions per second. For low reduced volume *v* = 0.25, we observed dynamic branched tubular networks with one to five junctions. At fixed Pe, the junction turnover rate *r* generally increases with increasing *ϕ*, as shown in Fig. 3 *A*. Interestingly, at fixed *ϕ*, *r* decreases with increasing Pe (Fig. 3 *B*), indicating that network reorganization occurs more frequently when filament mobility is lower. This trend is consistently observed across all *ϕ* ≥ 2.55% within the branched tubular regime.

The tubes emerge and retract with almost identical rates for all values of Pe and *ϕ*, as observed in the ratio *r*_*e*_/*r* ≈ 0.5 for all networks, as seen in Fig. 3 *C*. Therefore, despite the absence of steady-state behavior, nonequilibrium tubular networks maintain their complexity while dynamically reorganizing their structure. This novel feature arises from the combined effects of local vesicle remodeling driven by filament mobility and global large-scale shape transformations imposed by volume constraints. A comparison between the two panels in Fig. S4 highlights the significant influence of *v* on the dynamics of tubular networks. A slight increase in *v* from 0.25 to 0.3 leads to markedly slower reorganization of branched tubes, as reflected in lower values of *r*.

### Dynamic vesicle remodeling: Filament anisotropy

In addition to reduced volume and filament mobility, vesicle remodeling is strongly influenced by the mechanical properties of the filaments, particularly their length and stiffness, L and *χ*. The rescaled filament length L corresponds to the equilibrium contour length and is proportional to *N*_*bf*_-1, where *N*_*bf*_ is the fixed number of beads per filament in each simulation. However, the actual filament conformation also depends on interactions with the membrane boundary, collective alignment with neighboring filaments—especially at high concentrations—and active propulsion forces. As a result, the effective filament length depends not only on *χ* and L but also on *ϕ* and *v*. To capture this behavior, we use the average end-to-end distance—commonly referred to as the filament persistence length, P—as a more accurate measure to distinguish anisotropic (polar) filaments from isotropic (apolar) bead-like configurations.

**Figure 4:**
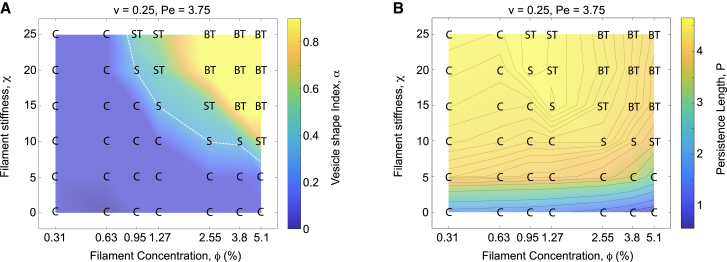
Vesicle morphologies under filament bending stiffness. (*A*) Morphological behavior of vesicles with active filaments at varying filament stiffness. Lower filament stiffness delays the transition from cups to sheets, sheet-tube structures, and eventually branched tubes at larger values of *ϕ*. Reduced stiffness also leads to the formation of crumpled filament aggregates, transforming branched tubes into cups for *χ* ≤ 5. The white dashed line indicates toroidal sheets predicted by the theoretical vesicle model (see materials and methods). (*B*) For small *χ*, crumpled filaments exhibit low persistence lengths, P, in cups, consistent with the vesicle shapes shown in (*A*).

At *v* = 0.25, we varied *χ* within the interval [0,25], with *χ* directly controlling the filament anisotropy via changing P. As expected, vesicle structures strongly depend on *χ*, and thus on filament anisotropy, as shown in Fig. 4 *A* for Pe = 3.75. For relatively stiff filaments with high *χ* = 25, we recover our previous results shown in Fig. 1 *B*. However, decreasing *χ*, which corresponds to more flexible and less straight filaments, drastically changes the morphological behavior of the vesicles. As *χ* decreases, the transition from cups to sheets, sheet-tubes, and eventually to branched tubes occurs at progressively higher values of *ϕ* (as indicated by the white dashed line denoting toroidal sheets). In other words, at higher filament concentrations, cup formation occurs at lower values of *χ*. This shift may result from the collective alignment of filaments at high concentrations, which delays the onset of cup formation until the filaments become sufficiently flexible. This observation also aligns with the right half of Fig. 4 *B*, where for relatively high *ϕ* > 1.27%, the filament persistence length P increases steadily with *ϕ*, as indicated by the isocurves of P.

To quantify how filament stiffness affects vesicle morphology, we varied *χ* within the interval [0,25] at fixed *v* = 0.25 and Pe = 3.75. As shown in Fig. 4 *A*, vesicle shapes undergo distinct transitions as *χ* decreases—from cups to sheets, sheet-tubes, and eventually to branched tubes. These transitions occur at progressively higher filament concentrations *ϕ*, suggesting that high *ϕ* delays the formation of cups. This shift likely results from the collective alignment of active filaments at high concentrations, which promotes more extended filament conformations even at lower stiffness. This interpretation is supported by Fig. 4 *B*, which shows that the filament persistence length P increases with *ϕ* at fixed *χ* for sufficiently large *ϕ* > 1.27% (see the right half of Fig. 4 *B*).

Branched tubes, predominantly observed at high *ϕ* and *χ*, gradually diminish as *χ* decreases from 25 and eventually vanish at *χ* = 10. For sufficiently low *χ* < 10, where filaments lose their anisotropy and crumple into apolar lumps, only cup-shaped vesicles are observed, even at high *ϕ*, as seen in Fig. 4 *A*. A similar behavior is observed for higher Pe values (Pe ≥ 7.5), as seen in Fig. S5. Consistently, corresponding values of P show apolar crumpled filaments for *χ* < 10, as seen in Fig. 4 *B*.

**Figure 5:**
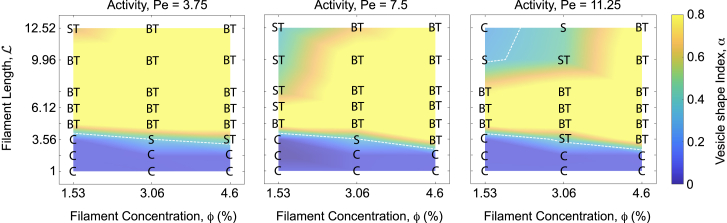
Vesicle morphologies under filament length. The morphological behavior of active vesicles exhibits a Pe-dependent response with varying filament length, L. Branched tubular structures (*yellow region*) appear only within a specific intermediate range of L, beyond which they transition into either cups or sheets. The white dashed lines indicate toroidal sheets predicted by the theoretical vesicle model at different values of *v* (see materials and methods). The intermediate branched tubular regime diminishes with increasing Pe. The simulations are performed at *v* = 0.25 and *χ* = 25.

For fixed *v* = 0.25 and *χ* = 25, we then varied *N*_*bf*_ within [1,10] to form filaments with 1≤L≤12.52 at different values of Pe = 3.75, 7.5, 11.25 (Fig. 5). The lower limit, L=1 with *N*_*bf*_ = 1, corresponds to active vesicles containing internal apolar spherical beads. The morphology diagram of active vesicles for varying *ϕ* and L features an intermediate branched tubular regime (yellow) surrounded by two nontubular regimes from below and above. Two transition regions (orange bands) separate the nontubular regimes from the branched tubular regime. At low L≲4, below the nearly horizontal transition band (orange), only cups are observed across different values of *ϕ* and Pe. As L increases, sheets and sheet-tubes emerge along and near the transition band, rapidly evolving into branched tubes beyond it. Interestingly, further increasing L results in the recovery of sheets and sheet-tubes and eventually cups at sufficiently high Pe beyond a second transition band (orange) as seen near the top left of the morphology diagrams. The lower transition band is closely approximated by the lower white dashed lines, which correspond to toroidal sheets. For sufficiently high Pe = 11.25, the upper transition band also encompasses the top white dashed line corresponding to toroidal sheets in the right panel of Fig. 5.

The transitions between the branched tubular regime and the surrounding nontubular regimes exhibit different behaviors depending on Pe. Although the bottom transition bands remain nearly horizontal and largely independent of *ϕ*, the top transition band shows a strong dependence on *ϕ*. Consequently, the initially small top nontubular regime, confined to low *ϕ* ≲ 2.5% at Pe = 3.75, expands with increasing Pe, reaching *ϕ* ≲ 4% at Pe = 11.25. As a result, the top transition band, which is absent at Pe = 3.75 and 7.5 due to the lack of sheets, emerges at Pe = 11.25 (right panel of Fig. 5). Therefore, vesicle shapes exhibit nonmonotonic behavior with respect to L, making their morphological transitions highly sensitive to Pe. This distinct sensitivity to Pe is unique to variations in L, in contrast to *χ* and *v*, where vesicle shapes show only a weak dependence on Pe. Our results indicate that tubular membranes, particularly dynamically reorganizing branched tubes, form within an intermediate range of L, which narrows as Pe increases. For very short filaments below this range and very long ones beyond it, branched tubes are not observed.

**Figure 6:**
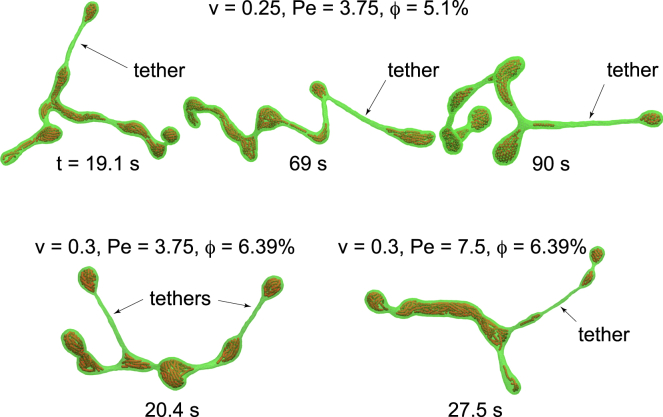
Membrane tethers form at high filament concentrations, *ϕ*. Short-lived tethers (lifetime ≲1 s) are observed almost exclusively on branched tubes with relatively large *ϕ*. These tethers have smaller diameters than their parent tubes.

In addition to different dynamic vesicle structures, we rarely observe short-lived membrane tethers with life times ≲1 s, which occur almost exclusively for branched tubular networks with relatively large *ϕ*, as seen in Fig. 6. These long, narrow tethers have diameters comparable to the filament width (i.e., the size of a single bead) and are typically thinner than the parent tube from which they extend. They usually broaden and reintegrate into the original tube within a short time. Similar tethers have been reported in vesicles with both normal and sticky active beads (22,30,40).

For less anisotropic filaments with relatively low P—corresponding to small L or *χ*—we also observe additional dynamically unstable cup-like variants. For instance, increasing *ϕ* at *v* = 0.25 leads to the formation of multicompartmentalized cups, as seen in Fig. S6 *A* and *B* (top-left panel). Another example is tube-cups, which consist of both cup-like and tubular sections and are observed at low L, as seen in Fig. S6 *B*, bottom panel. Like other unstable hybrid structures, such as sheet-tubes, branched tubes, and compartmentalized vesicles, these shapes also undergo continuous morphological reorganization, with shape units dynamically emerging, disappearing, and interconverting.

### Membrane tension

To explore how vesicle shapes and their dynamics relate to the large-scale effects of constrained volume and the local effects of internal active filaments, we calculate the average membrane tension $\bar{\lambda }$ in active vesicles (see materials and methods) and compare it to $\lambda_{0}=R^{2}k_{B}T/\left(\pi \sigma^{4}\right)$. The vesicles simulated here exhibit membrane tension in the range $\bar{\lambda }/\lambda_{0}=0.08-0.22$, corresponding to approximately 1–3 *μN*/*m* for a typical vesicle size of *R* = 1 *μm*. These vesicles are thus classified as floppy vesicles with almost negligible membrane tensions. Although $\bar{\lambda }$ appears to increase with *ϕ*, it remains constant across different values of Pe∈[3.75,15] (Fig. S8), suggesting that the shapes and dynamics of these floppy vesicles in the low-tension regime are not primarily governed by membrane tension.

## Conclusion

We explore dynamic shape remodeling of vesicles driven by the combined local effects of internally confined active filaments and the global effects of constrained volume, which regulates large-scale macroscopic shape transformations. In addition to the three fundamental vesicle shapes—tubes, sheets, and cups—which also occur in equilibrium in the absence of active elements, we observe intrinsically unstable hybrid structures such as branched tubes, sheet-tubes, pearled tubes, cup-tubes, and other compartmentalized morphologies like pear vesicles and sphere-tubes. These unstable hybrid vesicles are characterized by continuous dynamic restructuring, in which their constituent shape components interconvert while maintaining nearly constant proportions. For example, branched tubular networks reorganize by varying the number of tubular junctions while keeping the total tubular length nearly constant. Similarly, sheet-tubes restructure by changing the number of sheet-like and tubular segments while preserving the relative proportion of each component.

These dynamic structures are distinct from the nonequilibrium stationary shapes reported for similar vesicles containing confined active filaments in the absence of volume constraints (32). They also differ from the stationary vesicle shapes—typically tubes, sheets, and cups—observed under internal active Brownian particles and constrained volume, which transform into one another as particle mobility varies (27). For instance, the branched tubes we observe do not resemble the membrane tethers reported for vesicles containing both normal and sticky active particles (22,30,40). These tethers typically form at relatively high values of both Pe and *v* and are characterized by their very small diameter, comparable to the size of a single Brownian particle. In contrast, the branched tubular structures reported here exhibit a nearly uniform tube diameter that increases with *v*. Moreover, whereas membrane tethers are primarily observed at Pe ≲ 800, our branched tubes emerge at much lower values of Pe ≤ 15 (32). Notably, branched tubes are more abundant at low Pe and are limited to small volumes *v* ≤ 0.4. Although increasing Pe promotes tether formation, the occurrence of branched tubes increases with decreasing Pe.

Remarkably, the dynamic reorganization of these unstable hybrid structures accelerates as Pe decreases. Moreover, at relatively small Pe, the vesicles exhibit low membrane tensions that remain nearly constant across different values of Pe. This suggests that the dynamic restructuring of these floppy vesicles is governed more strongly by volume constraints than by filament mobility. Overall, the regime of low filament mobility inside floppy vesicles gives rise to unstable hybrid morphologies—such as branched tubes, sheet-tubes, and pearled vesicles—that continuously reorganize their shapes at rates that increase as Pe decreases.

We demonstrate that both the formation and dynamics of these unstable hybrid structures strongly depend on the anisotropic nature of the active filaments. Reducing filament stiffness at fixed length, or decreasing filament length (i.e., number of beads) at fixed stiffness, results in less anisotropic filaments with smaller P. Dynamically reorganizing hybrid vesicles—such as branched tubes and sheet-tubes—become less abundant as filament anisotropy decreases, and they eventually vanish when filaments become fully crumpled or reduce to single-bead isotropic active particles (spherical particles), giving rise to cup-like vesicles. Our results thus highlight the importance of the filamentous nature of the cytoskeletal network in forming nonequilibrium dynamic structures of the cellular membrane. In particular, the dynamic branched tubes and sheet-tubes resemble cellular protrusions such as filopodia and lamellipodia, as well as the membrane morphologies observed in organelles like the endoplasmic reticulum and mitochondria.

## Acknowledgments

A.H.B. acknowledges support from the 10.13039/501100004189Max Planck Society within the framework of Max Planck Partner Group and from the 10.13039/100004410European Molecular Biology Organization, grant 10.13039/501100003043EMBO IG 5032.

## Author contributions

A.H.B. designed research; A.K.S. and A.H.B. performed simulations; A.K.S. and A.H.B. analyzed the data. A.H.B. wrote the manuscript with assistance from A.K.S.

## Declaration of interests

The authors declare no competing interests.

## Supporting material

Document S1. Figures S1–S8 and Table S1

Document S2. Article plus supporting material
