# Supplementary material for: Dynamic shape remodeling of vesicles by internal active filaments
Source: Biophys J. 2025 Oct 21;124(23):4281–92. doi: 10.1016/j.bpj.2025.10.021 (PMC12709404; doi:10.1016/j.bpj.2025.10.021)
Supplement: Document S1. Figures S1–S8 and Table S1 [file mmc1.pdf]

Biophysical Journal, Volume *124*

Supplemental information

Dynamic shape remodeling of vesicles by internal active filaments

Arash Karaei Shiraz and Amir H. Bahrami

Supplementary Information
for
Dynamic shape remodeling of vesicles by internal active filaments

Arash Karaei Shiraz and Amir H. Bahrami*

Living Matter and Biophysics, UNAM — National Nanotechnology Research Center and Institute of Materials Science and Nanotechnology, Bilkent University, N. 30, 1598 St., 06800 Ankara, Turkey

E-mail: bahrami@unam.bilkent.edu.tr

This Supplementary Information contains:

1. Supplementary Figures:

Fig. S1: Vesicle morphologies formed by internal active filaments.

Fig. S2: Morphology diagram of active vesicles

Fig. S3: Temporal evolution of dynamically reorganizing active vesicles

Fig. S4: Dynamic reorganization of highly branched tubular networks

Fig. S5: Morphological behavior of vesicles with active filaments at varying χ

Fig. S6: Vesicle morphologies for varying $\mathcal{L}$ and χ

Fig. S7: Theoretical vesicle models

Fig. S8: Membrane tension in active vesicles

2. Supplementary Table:

Tab. S1: Principal parameters

3. Legends of Supplementary Videos:

Movie S1: Dynamic shape remodeling of a branched tube at low volume

Movie S2: Dynamic shape remodeling of a branched tube at intermediate volume

Movie S3: Dynamic shape remodeling of a sheet-tube

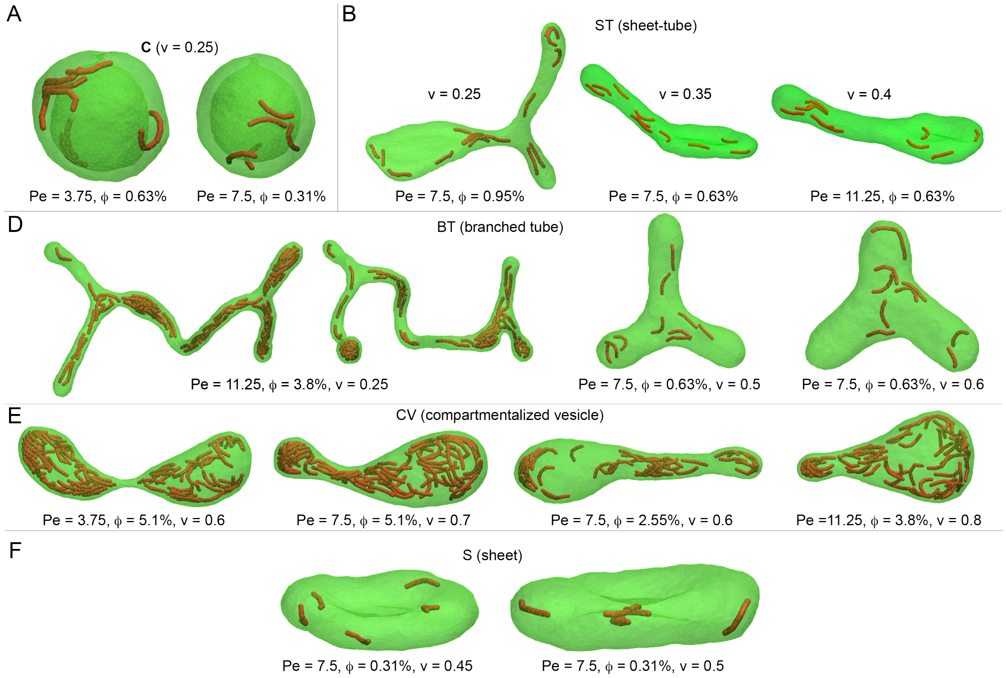

Figure S1. Vesicle morphologies formed by internal active filaments. Different vesicle shapes are shown with their corresponding simulations times from left to right in each panel. (A) Cups (40 s and 50 s). (B) Sheet-tubes (50 s for all three snapshots). (D) Branched tubes at low and high volumes (36.8 s, 41.7 s, 50 s, and 20.4 s). (E) Compartmentalized vesicles including pearled tube (left) and pear-shaped vesicles (right) (33.6 s, 50 s, 50 s, and 50 s). (F) Sheets (50 s for both snapshots).

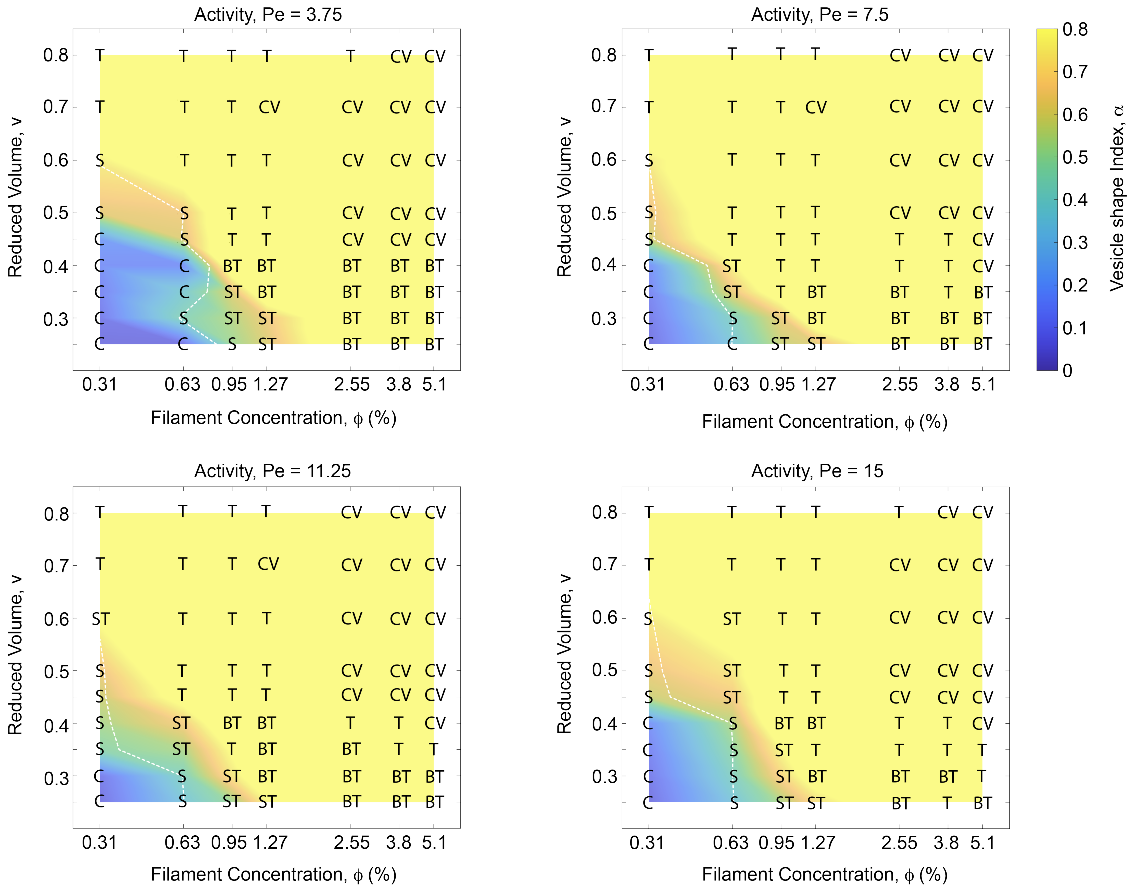

Figure S2. Morphology diagram of active vesicles. Vesicle shapes with varying ϕ, v, and Pe for relatively stiff filaments ($\chi = 25$) of intermediate length ($\mathcal{L} = 6.12$). Vesicle shapes are characterized by the shape index α, which is based on membrane asymmetry (Δa) and is color-mapped onto the shape diagram. The white dashed line indicates toroidal sheets predicted by the theoretical vesicle model at different values of v (see Methods).

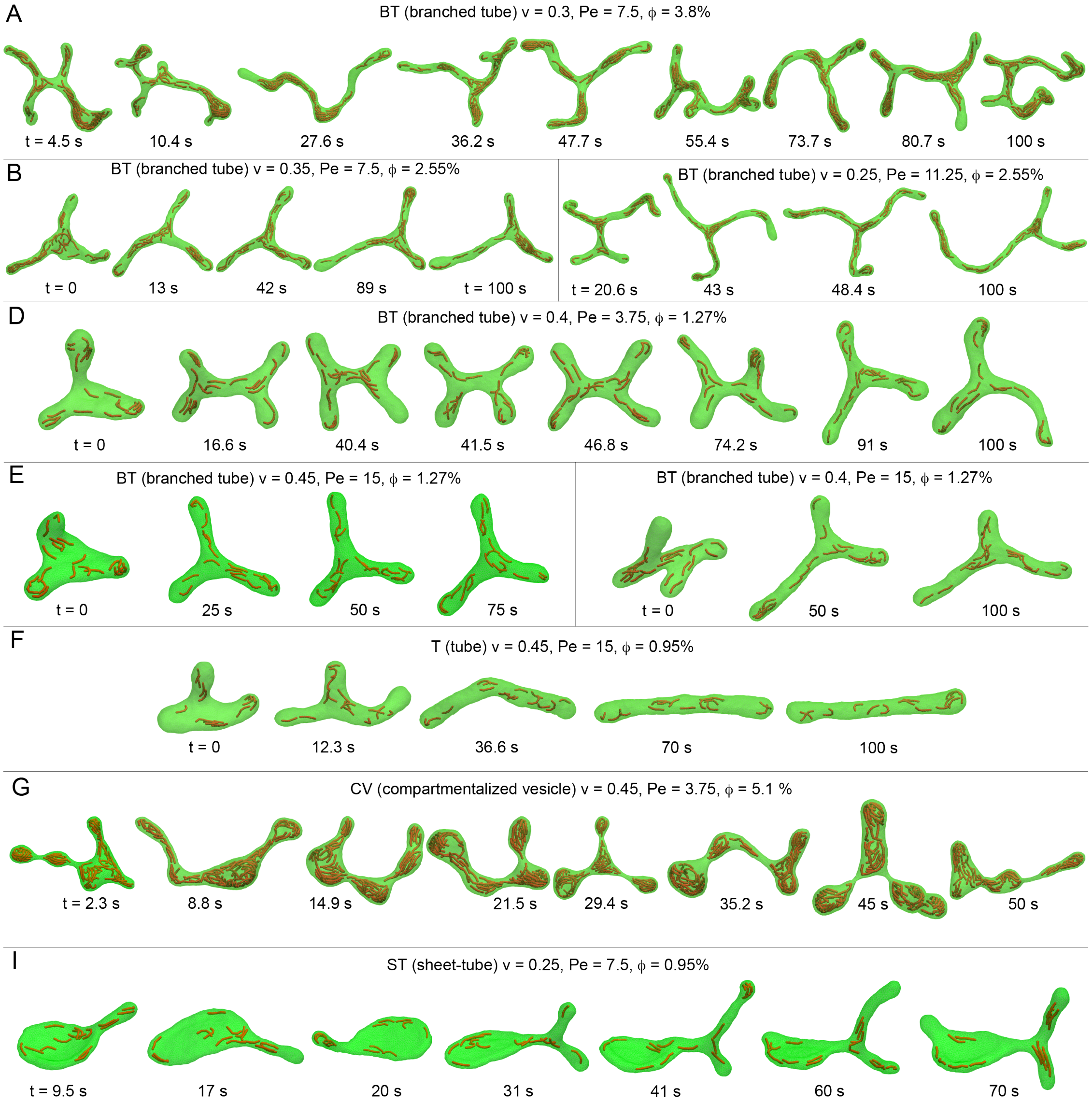

Figure S3. Temporal evolution of dynamically reorganizing active vesicles. (A-E) Branched tubular networks with different values of v, continuously reorganize their structures by extending and retracting tubes, forming a variable number of three-way tubular junctions. (F) Unstable short-lived branched tube at relatively large $v = 0.45$ transforming to a stable tube. (G) Temporal evolution of a highly dynamic compartmentalized vesicle, composed of restructuring compartments, at a relatively large ϕ over 50 seconds. (I) A sheet-tube (ST) dynamically reorganizes its shape by transitioning from one to two tubular segments over 60 seconds.

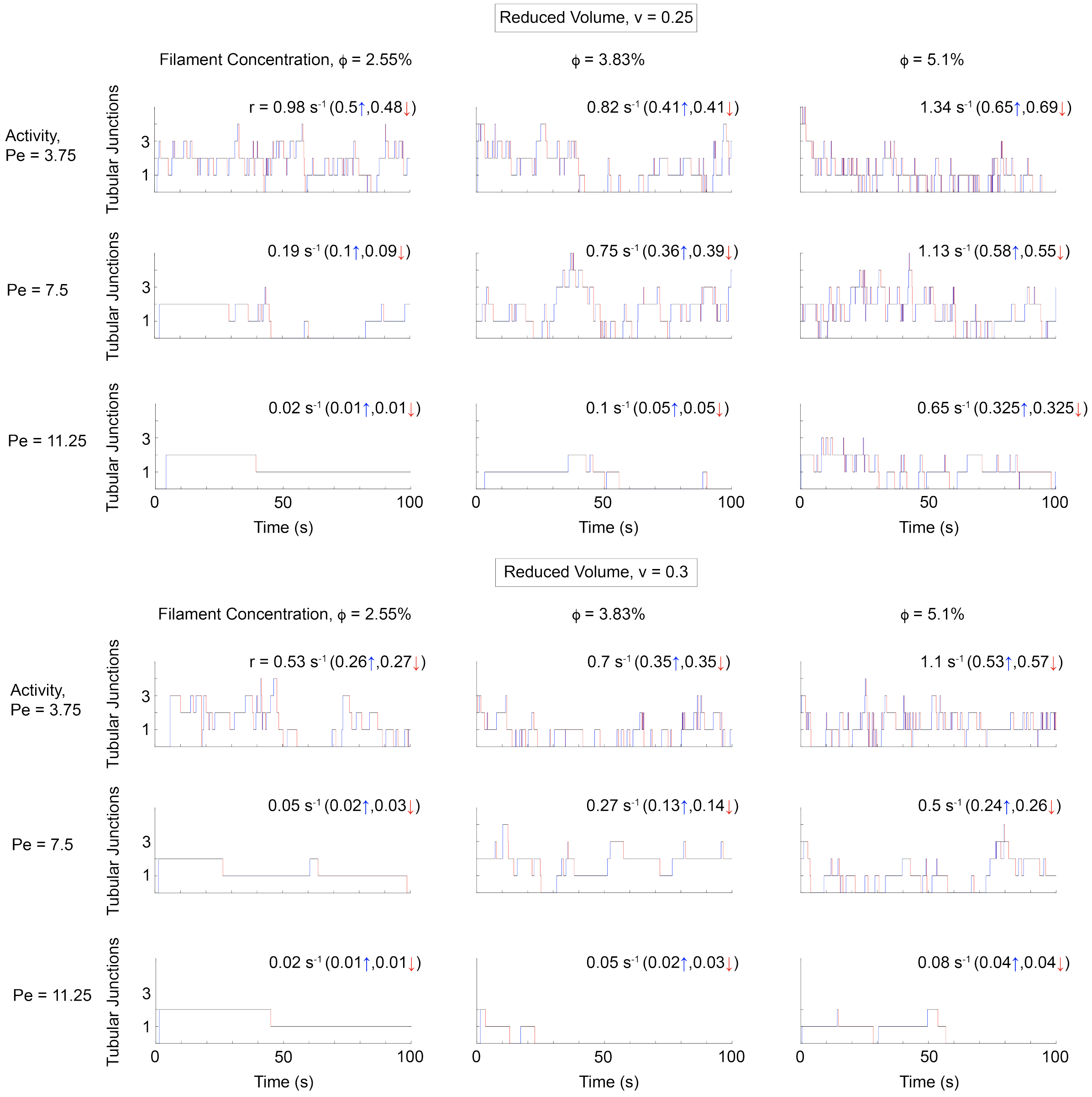

Figure S4. Dynamic reorganization of highly branched tubular networks. Temporal evolution of the number of three-way tubular junctions for different values of Pe and ϕ at $v = 0.25$ and $v = 0.3$. The total rate of change in the number of tubular junctions is given by the sum of the tube formation rate (blue lines) and the tube retraction rate (red lines). The rate of change in tubular junctions increases with ϕ for a fixed Pe, while it decreases with Pe at a constant ϕ. All branched tubes exhibit nearly identical formation and retraction rates, implying that tube formation and retraction occur with almost equal frequency.

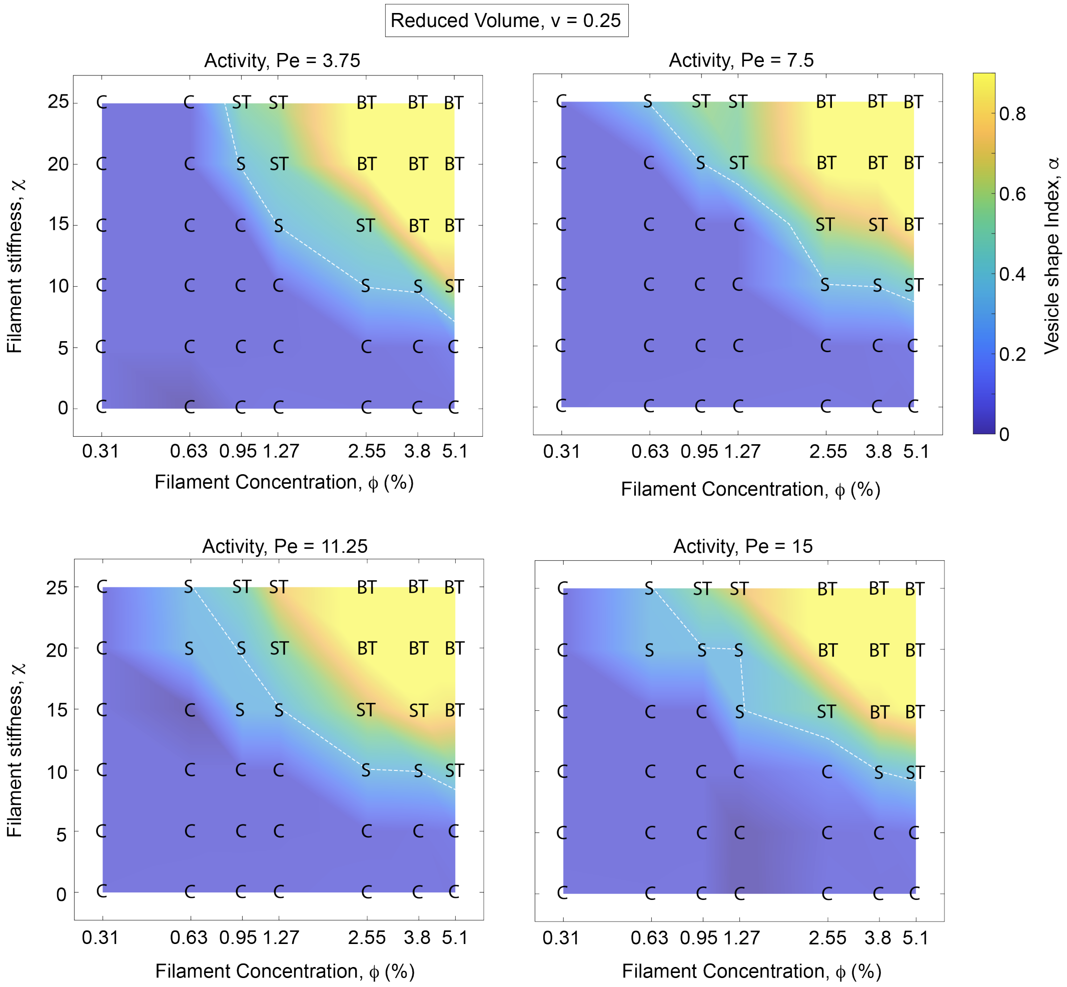

Figure S5. Morphological behavior of vesicles with active filaments at varying χ. Vesicle shapes are obtained for $v = 0.25$ and different values of Pe. Lower filament stiffness delays the transition from cups to sheets, sheet-tube structures, and eventually to branched tubes at higher values of ϕ. Reduced stiffness also leads to the formation of crumpled filaments, transforming branched tubes into cups for $\chi \leq 5$. The white dashed line indicates toroidal sheets predicted by the theoretical vesicle model at different values of v (see Methods). Vesicle morphologies show only weak sensitivity to Pe.

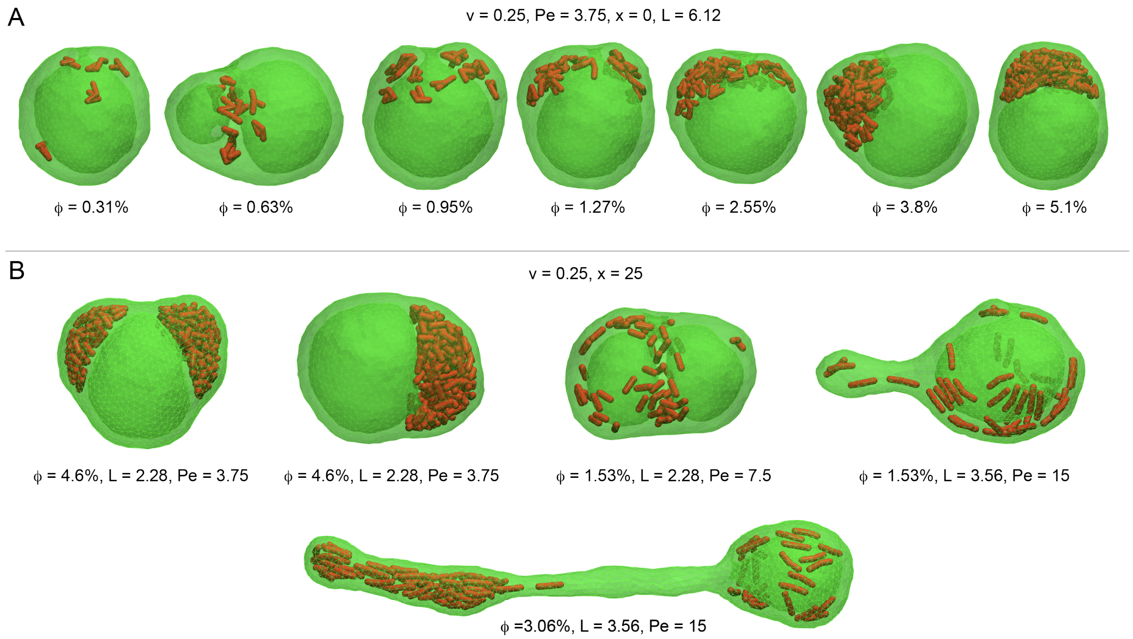

Figure S6. Vesicle morphologies for varying $\mathcal{L}$ and χ. (A) A variety of cup-like vesicles formed by active filaments with negligible bending stiffness (χ), shown for different values of ϕ at fixed $v = 0.25$. (B) Distinct cup-like variants, including compartmentalized cups, double-cups, and cup-tubes (comprising both cup-like and tubular segments), observed for stiff active filaments with varying filament lengths $\mathcal{L}$, ranging from $\mathcal{L} = 1$ (corresponding to spherical active beads) to $\mathcal{L} > 1$ (extended active filaments). All snapshots correspond to $t = 50$ s.

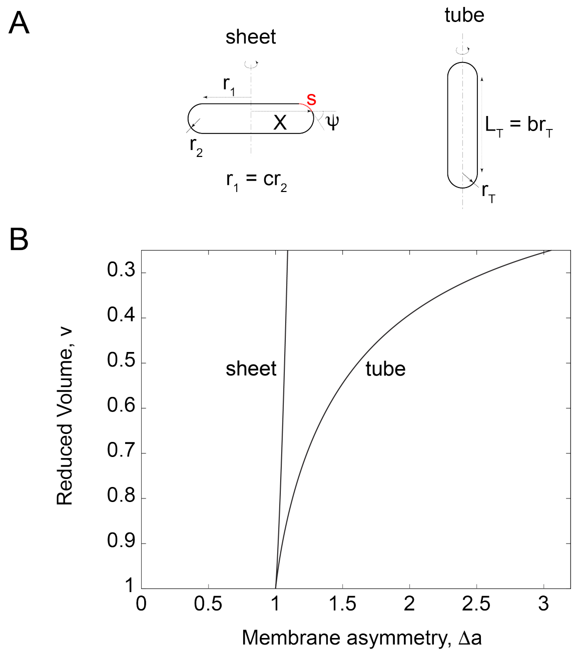

Figure S7. Theoretical vesicle models. (A) Schematics of axisymmetric vesicle shapes: a toroidal sheet (left) and cylindrical tubes (right). (B) Reduced volume of toroidal sheets and cylindrical tubes plotted as a function of membrane asymmetry, Δa. A straight line is fitted to the toroidal sheet data, defining the white dashed lines in Figs. 1, 4, 5, S2, and S5. The vesicle shape index, $\alpha = \Delta a / \Delta a_T$, is calculated using the membrane asymmetry of the cylindrical tube, Δa_T (right curve), at each value of v.

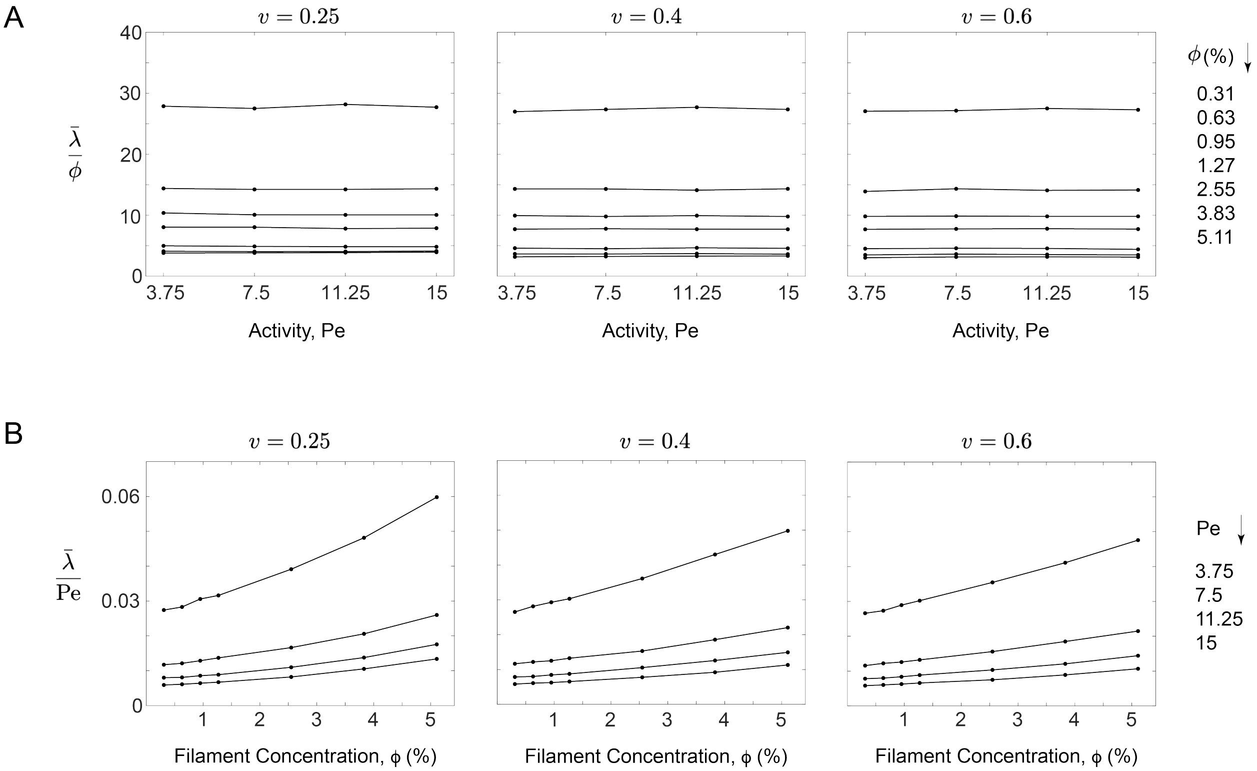

Figure S8. Membrane tension in active vesicles. (A) Average membrane tension as a function of Pe for different values of ϕ. (B) Average membrane tension as a function of ϕ for different values of Pe.

Table S1: Principal parameters

Parameters	Model units	Physical units
	Basic properties	
reference vesicle radius R	0.62035	1 μm
time scale $\tau = \gamma R^2/\kappa$	1.925×10^5	0.1925 s
thermal energy unit $k_B T$	1	$4.14{\times}10^{-21}$ J
	System parameters	
Péclet number $\text{Pe} = \sigma f_p/k_B\mathbb{T}$	3.75 - 15	3.75 - 15
number of beads per filament N_{bf}	1 - 10	1 - 10
number of beads $N_b = N_f \times N_{bf}$	25 - 400	25 - 400
filament concentration $\phi = N_f N_{bf}(\sigma/2R)^3$	0.31 - 6.4	0.31 - 6.4
desired vesicle volume V_0	0.25 - 0.8	1.05 - 3.35 μm^3
filament length $\mathcal{L} = (N_b - 1)r_0 + \sigma$	1 - 12.52	1 - 12.52
filament stiffness $\chi = \kappa_f/\kappa$	0 - 25	0 - 25
	Vesicle properties	
number of vertices N_v	2562	2562
bending rigidity κ	$20k_B\mathbb{T}$	8.28×10^{-20} J
equilibrium bond length l_0	$4R\sqrt{\frac{\pi}{N_t\sqrt{3}}}$	0.075 μm
potential cutoff r_r	$0.95l_0$	0.0715 μm
potential cutoff r_a	$1.05l_0$	0.079 μm
bond stiffness k_b	$11.54k_B\mathbb{T}/R^2$	4.78×10^{-17} J.μm^{-2}
desired vesicle area A_0	$4\pi R^2$	12.57 μm^2
area conservation coefficient k_A	$3.72 \times 10^5 k_B\mathbb{T}/R^2$	1.54×10^{-15} J.μm^{-2}
volume conservation coefficient k_V	$2.38 \times 10^6 k_B\mathbb{T}/R^3$	9.88×10^{-15} J.μm^{-3}
friction coefficient γ	$20k_B\mathbb{T}\tau/R^2$	1.59×10^{-20} J.sμm^{-2}
	Active filament properties	
effective bead diameter σ	$R/10$	0.1 μm
equilibrium filament bond length r_0	1.28σ	0.129 μm
potential coefficient k_{bb}	$1.15 \times 10^6 k_B\mathbb{T}/R^2$	4.78×10^{-15} J.μm^{-2}
filament bond stiffness k_{fb}	$1.15 \times 10^4 k_B\mathbb{T}/R^2$	4.78×10^{-17} J.μm^{-2}

Movie S1. **Dynamic shape remodeling of a branched tube at low volume**. Dynamic shape remodeling of the branched tube at $v = 0.25$ over 100 seconds, driven by internal active filaments. The movie corresponds to the snapshots shown in Fig. 2A. At the smallest filament mobility (Pe = 3.75), this branched tube rapidly reorganizes its structure by dynamically varying the number of tubular junctions, as illustrated in the top-left panel of Fig. 3A.

Movie S2. Dynamic shape remodeling of a branched tube at intermediate volume. Dynamic shape remodeling of the branched tube at $v = 0.35$ over 100 seconds, as shown in the snapshots in Fig. 2B. Compared to the branched tube in Movie. S1, which has a smaller v, this tube—with the same Pe—exhibits wider diameters and slower dynamic reorganization.

Movie S3. Dynamic shape remodeling of a sheet-tube. Dynamic shape transitions of a sheet-tube at $v = 0.25$ over 100 seconds, corresponding to the snapshots in Fig. 2D. Despite continuous interconversion between sheet-like and tubular sections, the area proportion of each component remains nearly constant. Although the number of tubes and their individual lengths fluctuate, both the total tubular length and the tube diameter remain approximately unchanged.
